# Diagnostic Accuracy of Fecal Calprotectin in Discriminating Organic-Inflammatory Gastrointestinal Diseases and Functional Gastrointestinal Disorders in Older Patients

**DOI:** 10.3390/jpm14030227

**Published:** 2024-02-21

**Authors:** Antonella Gallo, Marcello Covino, Silvia Baroni, Sara Camilli, Francesca Ibba, Silvia Andaloro, Maria Chiara Agnitelli, Fiammetta Maria Rognoni, Francesco Landi, Massimo Montalto

**Affiliations:** 1 Department of Geriatrics, Orthopedics and Rheumatology, Fondazione Policlinico Universitario “A. Gemelli”, IRCCS, 00168 Rome, Italy; 2 Department of Emergency Medicine, Università Cattolica del Sacro Cuore, 00168 Rome, Italy; marcello.covino@policlinicogemelli.it; 3 Fondazione Policlinico Universitario “Agostino Gemelli”, IRCCS, 00168 Rome, Italy; silvia.baroni@policlinicogemelli.it (S.B.); francesco.landi@unicatt.it (F.L.); massimo.montalto@unicatt.it (M.M.); 4 Department of Chemistry, Biochemistry and Clinical Molecular Biology, Università Cattolica del Sacro Cuore, 00168 Rome, Italy; 5 Department of Geriatrics, Orthopedics and Rheumatology, Università Cattolica del Sacro Cuore, 00168 Rome, Italysilvia.andaloro01@icatt.it (S.A.); agnitelli01@icatt.it (M.C.A.);

**Keywords:** fecal calprotectin, elderly, inflammatory bowel diseases

## Abstract

Fecal calprotectin (FC) has been largely recognized as a surrogate marker of intestinal neutrophilic inflammation, very reliable in distinguishing between inflammatory bowel diseases and functional gastrointestinal (GI) disorders. Aging has been suggested to influence FC results and their diagnostic accuracy; however, no studies are specifically targeted on this focus. In a retrospective study, we evaluated the eventual age-differences of the diagnostic accuracy of FC in discriminating between organic-inflammatory GI diseases and functional GI disorders. In 573 younger and 172 older (≥65 years) subjects undergoing an FC assay, we found that the latter showed higher median FC values, 72 (25–260) µg/g vs. 47 (25–165) µg/g (*p* < 0.01). Younger patients were more commonly affected by IBDs, while colorectal cancer and high-risk polyps, infective colitis, and diverticular disease represented the most common findings in the older subgroup. However, the estimated optimum FC threshold in discriminating between organic-inflammatory GI diseases and functional GI disorders was quite similar between the two groups (109 μg/g for the younger subgroup and 98 μg/g for the older subgroup), maintaining a very high specificity. In conclusion, we show that FC also represents a very specific test for intestinal inflammation in older patients, at similar threshold levels to younger subjects.

## 1. Introduction

Patients presenting for medical attention with gastrointestinal (GI) symptoms often represent a real challenge and can be difficult to assess. In particular, the relatively non-specific clinical manifestations of gastrointestinal disease can make it difficult for clinicians to distinguish between functional gastrointestinal disorders (FGID) and organic gastrointestinal disease (OGID), especially in patients presenting without rectal bleeding or other alarming symptoms [1].

The primary distinction between FGID and OGID lies in the inflammatory nature of the latter [2]. The diagnostic gold standard for identifying bowel inflammation consists of endoscopy with biopsies and histology. However, colonoscopy is an invasive and expensive procedure, which might not be necessary for those patients presenting with abdominal symptoms who actually have a functional disorder. Hence, there has been considerable advocacy for a non-invasive and easily quantifiable test that can predict the necessity of an invasive investigation [3].

Calprotectin is a calcium-binding cytosolic protein composed of two subunits (S100A8 and S100A9) primarily secreted by neutrophils, playing a crucial role in the innate immune response [3,4,5]. Fecal calprotectin (FC) has been largely recognized as a surrogate marker of intestinal neutrophilic inflammation [4]. Its clinical utility relies on its clinical and laboratory characteristics: it demonstrates a high negative predictive value, remains resistant to degradation caused by digestive enzymes, and can be stored at room temperature for up to seven days [5].

Numerous studies have shown significantly increased FC levels among patients with active inflammatory bowel disease compared to control groups [6,7,8,9]. Moreover, it represents a reliable marker in distinguishing between inflammatory bowel disease (IBD) and irritable bowel syndrome (IBS) [7,8,9], as well as in the management of IBDs, because of the close correlation with the mucosal healing process [3].

Although most of the evidence has focused on the role of FC in IBD diagnosis and management, slightly increased FC levels have been reported in patients with various inflammatory conditions affecting the lower gastrointestinal tract beyond IBD [10,11].

A significant challenge in the laboratory assessment of fecal calprotectin lies in establishing the upper limit for healthy individuals, ranging from 50 μg/g to 100 μg/g in most available studies [12,13,14].

Various factors, such as age and obesity, have been identified as potential influencers of FC test results [15,16]. However, only a few previous studies have proposed to modify the laboratory ranges for older individuals [15,16], mainly based on the hypothesis that age-related changes in the immune system, alterations in lifestyle, and diet could influence inflammatory responses in the gastrointestinal mucosa.

Currently, there is a lack of studies specifically targeting the diagnostic accuracy of FC. Our study seeks to explore any eventual differences between younger and older subjects concerning the diagnostic accuracy of FC in discriminating between organic-inflammatory GI diseases and functional GI disorders.

## 2. Patients and Methods

### 2.1. Patient Selection and Data Collection

This was a retrospective single-center study performed at a tertiary care center (Fondazione Policlinico Gemelli, Rome, Italy). Patients were selected using a Fecal Calprotectin Register (FCR), a record kept by our Biochemistry Department. This register contains patient ID, date of birth, and FC concentration for all of the samples analyzed.

Patients were included if they had their first FC from January 2015 to at least June 2022, to allow sufficient follow-up time. Where multiple FC samples were listed for the same patient, we included in the final analysis the closest FC value to the date of the final diagnosis or assessment of disease activity. Only FC samples performed within 30 days of an endoscopic or radiologic diagnosis of organic-inflammatory GI disease were included in the final analysis. In cases of functional GI disorders, time exceeding 30 days from the FC assay to the final diagnosis did not represent an exclusion criterion.

Data were collected retrospectively by reviewing electronic patient records, including all endoscopic and radiological investigations, clinic appointments, and hospital admissions. Patients were followed up for at least one year after the first presentation, and all re-presentations and subsequent diagnoses were noted.

The recorded parameters included age, gender, FC level, and date of the sample, as well as overall comorbidity status assessed by the Charlson Comorbidity Index (CCI). The indication to undergo an FC assay was extrapolated by reviewing all available medical records and identified as either a) main complaint (diarrhea, rectal bleeding, abdominal pain), or b) regular follow-up in subjects with a known IBD diagnosis. Specifically, for IBD patients, only those with a stated diagnosis of active/in remission IBD at the time of the FC assay were included in the study. The final diagnosis was recorded by reviewing the available records, including instrumental investigations performed and blood results. Records with insufficient data to identify both the reason for the FC assay and the final diagnosis were excluded.

Diagnoses were firstly recorded and then assigned to one of two groups for the final analysis: “organic-inflammatory GI diseases” and “functional GI disorders”. Patients with a documented organic-inflammatory GI disease were further divided into those with active IBD and patients with other organic-inflammatory GI diseases except for IBD, known to be associated with increased levels of FC [3,10]. In particular, they included colorectal cancer and high-risk polyp [3], active colon mucosal inflammation not including IBD [3], infective colitis, ischemic colitis, diverticulitis (complicated/uncomplicated acute diverticulitis, symptomatic uncomplicated diverticular disease (SUDD) [17], upper endoscopy inflammatory pathological findings (LA class C or D esophagitis +/− esophageal ulcer, severe/hemorrhagic gastritis or duodenitis with mucosal breaks or erosions [3].

In the second group, we included those patients not fitting with criteria for organic-inflammatory GI diseases. They were classified as follows based on the Rome IV criteria: (a) subjects with a specified FGID (IBS, functional constipation, functional diarrhea, or functional bloating with or without distension); (b) subjects with unspecified functional bowel disorders (FBD) [18]. In any case, we assessed the combination of normal gastrointestinal endoscopy or radiological findings (normal finding/uncomplicated hemorrhoids/asymptomatic diverticulosis), non-adenomatous polyp [3,19], and the absence of organic-inflammatory GI disease after at least a 12 month-follow-up from the time of the index test.

Blood results, including white blood cell counts and C-reactive proteins, were recorded if performed within a week of the FC assay.

### 2.2. FC Assay Technique

Stool samples were collected in suitable containers and frozen at −80 °C until the assay. They were then thawed and prepared using the LIAISON^®^ Q.S.E.T. Device Plus (REF 319060) containing a specific extraction buffer for the calprotectin assay and then shaken for 40 min. The analysis was performed on the LIAISON^®^ XL Analyzer using the DiaSorin LIAISON^®^ Calprotectin in chemiluminescence (CLIA) kit, sandwich-type, employing 2 monoclonal antibodies for the quantitative determination of calprotectin. In this system, the light signal is measured by a photomultiplier in relative light units (RLU) and is proportional to the concentration of calprotectin present in the samples based on the values obtained from the calibration curve. The measurement range of the method is 5–800 µg/g and the normality cut-off is 50 µg/g. The intra-assay and inter-assay CV are <8%.

### 2.3. Statistical Analysis

Categorical variables are presented as absolute numbers and percentages; continuous variables are presented as the median (interquartile range). Categorical variables were compared by a Chi-square test or Fisher’s exact test as appropriate. Continuous variables were compared by the Mann–Whitney U test. The overall accuracy of the calprotectin value to distinguish organic intestinal diseases was evaluated by the receiver operating characteristics (ROC) area under the curve (AUROC). The comparison between ROC curves in non-paired samples was performed by the Z statistic. The Youden index J was used to determine the best cut-off value for discrimination in the general population and the group above and below 65 years of age. Positive and negative predictive values and 95% confidence intervals were calculated for different cut-off levels of the calprotectin value.

The significance value was set at 0.05 (two-sided) in all of the analyses. The analysis was conducted by SPSS version 25 (IBM, Armonk, NY, USA) and MedCalc^®^ Statistical Software version 18 (MedCalc Software Ltd., Ostend, Belgium; https://www.medcalc.org (accessed on 30 December 2023)).

## 3. Results

### 3.1. Sample Study

Our FCR, relative to the study period, contains the data of 13,147 FC samples from 10,304 patients. We excluded 6270 incomplete records lacking a final diagnosis. An additional 1763 records were excluded as they were relative to an FC assay performed over 30 days of a radiological/endoscopic evaluation leading to a final diagnosis of organic-inflammatory GI disease.

**Figure 1:**
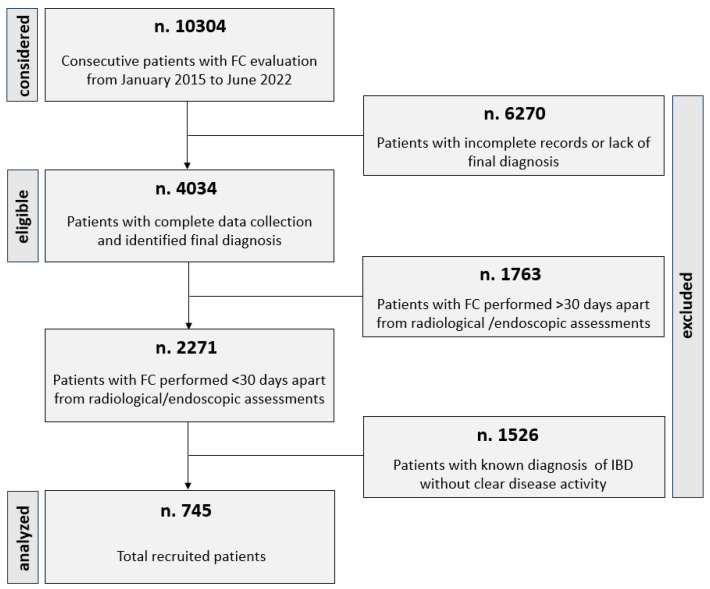
Selection of the final cohort.

Moreover, 1526 records were excluded as they were related to FC assays in subjects with a known IBD diagnosis but lacked a contextual assessment of disease activity. Thus, our final study cohort included 745 subjects (males 316, 429 females) with a median age of 48 (33–63) years (Figure 1).

In particular, 573 (76.9%) were younger than 65 years, while 172 (23.1%) were older than 65 years. Demographic and main clinical data are presented in Table 1, both for the total cohort and the different subgroups stratified by age.

Notably, older patients exhibited higher median fecal calprotectin values than younger subjects: 72 (25–260) µg/g vs. 47 (25–165) µg/g (*p* < 0.01). Younger subjects were more frequently affected by IBDs, while colorectal cancer and high-risk polyps, infective colitis, and diverticular disease represented the most common findings in the older subgroup with an organic-inflammatory GI diseases (*p* < 0.01 for all comparisons, Table 1).

### 3.2. Organic-Inflammatory GI Diseases and Functional GI Disorders

Upon reviewing all the available medical records, we found that 256 (34.3%) subjects were diagnosed with an organic-inflammatory GI disease (Table 2). Since IBD represents the most common diagnosis, with a significantly higher prevalence in the younger subgroup (Table 1), the overall percentage of organic-inflammatory GI diseases was lower in the older group (20% vs. 28.9%, *p* < 0.01). Diarrhea and rectal bleeding were more frequent in the subgroup of patients with a final organic-inflammatory GI disease, while abdominal pain was more common in those who received a final diagnosis of functional GI disorders (Table 2, *p* < 0.01 for all comparisons).

As expected, median fecal calprotectin, as well as median white blood cell counts and C-reactive protein levels, resulted significantly higher in those with a final diagnosis of organic-inflammatory GI diseases vs. functional GI disorder (Table 2, *p* < 0.01 for all comparisons).

### 3.3. Diagnostic Accuracy of Fecal Calprotectin in Discriminating Organic-Inflammatory GI Diseases vs. Functional GI Disorders According to Age

Table 3 shows the sensitivities, specificities, and positive and negative predictive values for FC in discriminating organic-inflammatory GI diseases vs. functional GI disorders in the total cohort and in the two different age subgroups at different thresholds. By using Youden’s formula, we estimated the optimum calprotectin threshold as 102 μg/g for the total cohort, with a sensitivity of 95.1 (95% CI 92.8–96.8) and specificity of 85.2 (95% CI 80.2–89.3). The best threshold FC level was quite similar between the two subgroups; in particular; it was 109 μg/g for the younger subgroup, with a sensitivity of 95.9 (95% CI 93.4–97.66) and specificity of 86.3 (95% CI 80.4–90.9), and 98 μg/g for the older subgroup, with a sensitivity of 93.9 (95% CI 87.1–97.7) and specificity of 82.4 (71.8–90.3).

**Figure 2:**
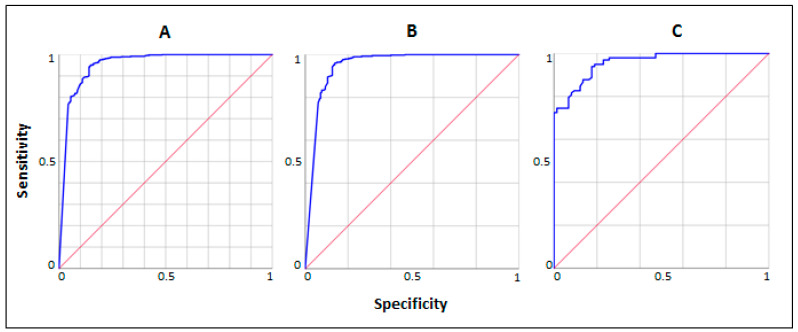
Receiver operating characteristic analysis for FC in discriminating organic-inflammatory GI diseases (including IBDs) vs. functional GI disorders in the total cohort and in the two age subgroups. (**A**) Total cohort; (**B**) Older patients; (**C**) Younger patients. GI: gastrointestinal. IBDs: inflammatory bowel diseases.

Receiver operating characteristic (ROC) analysis revealed an area under the curve (AUC) for FC of 0.95 [95% CI 0.88–0.98] for discriminating organic-inflammatory GI diseases (including IBD) vs. functional GI disorders in the total cohort (Figure 2A), 0.96 [95% CI 0.92–0.98] in the subgroup of older patients (Figure 2B) and 0.95 [95% CI 0.93–0.97] in the subgroup of younger patients (Figure 2C).

### 3.4. FC in Discriminating Organic-Inflammatory GI Diseases, except for IBDs, vs. Functional Disorders According to Age

Table 4 displays the main features of our cohort, according to a final diagnosis of GI organic-inflammatory GI diseases vs. functional GI disorders, not including IBDs. The detailed description of the main organic-inflammatory GI diseases except for IBDs has already been reported in Table 1, regarding both the total cohort and the two subgroups divided by age.

Also in this case, as expected, FC was significantly higher in those who showed an organic-inflammatory GI diseases, with respect to those who did not (*p* < 0.011, Table 4).

Table 5 shows the sensitivities, specificities, and positive and negative predictive values for FC in discriminating organic-inflammatory GI diseases vs. functional GI disorders in the total cohort and in the two different age subgroups at different thresholds, excluding those subjects affected by IBDs. Using Youden’s formula, we estimated the optimum calprotectin threshold as ≤70 μg/g for the total cohort, ≤102 μg/g in the younger subjects, and ≤45 μg/g in the older subgroup.

**Figure 3:**
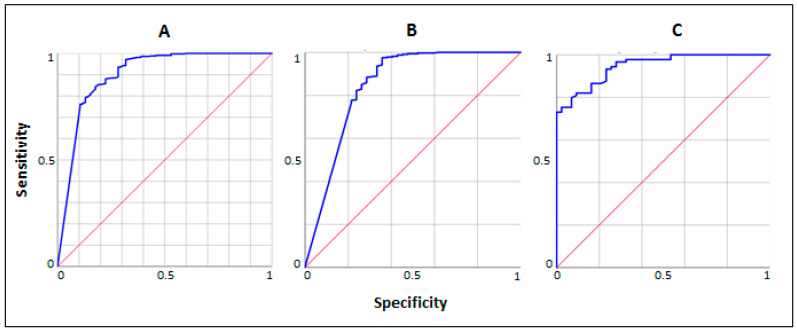
Receiver operating characteristic analysis for FC in discriminating organic-inflammatory GI diseases (not including IBD) vs. functional GI disorders in the total cohort and in the two age subgroups. (**A**) Total cohort; (**B**) Older patients; (**C**) Younger patients. GI: gastrointestinal. IBDs: inflammatory bowel diseases.

Receiver operating characteristic (ROC) analysis revealed an area under the curve (AUC) for FC of 0.90 [95% CI 0.87–0.93] for discrimination of organic-inflammatory GI diseases (not including IBDs) vs. functional GI disorders in the total cohort (Figure 3A), 0.96 [95% CI 0.93–0.97] in the subgroup of younger patients (Figure 3B) and 0.95 [95% CI 0.89–0.98] in the subgroup of older patients (Figure 3C).

## 4. Discussion

Our work represents the first attempt to evaluate the diagnostic accuracy of FC in a cohort of older adults.

Up to now, there are only a limited number of reports suggesting different age-related threshold levels of FC, with the few existing ones focused on healthy patients [16]. About ten years ago, Joshi et al. investigated the eventual age-related differences in various gastrointestinal inflammatory markers, including FC, in a group of 132 healthy subjects, aged from 2 to 86 years. They found that the median concentration in the 20 subjects over 60 years was 27 µg/g, against 22 µg/g in the 85 subjects aged 10–59 years and 34 µg/g in the 27 children aged 2–9 years [16]. Based on their results, they extrapolated different age-related reference ranges; in particular, for children between 2 and 9 years, the suggested cut-off was 166 µg/g, compared to 51 µg/g for the population aged 10–59 years and <112 µg/g in healthy patients over 60 years [16]. However, the definition of “healthy subjects” was only derived from history taking, and no endoscopic investigations were performed to confirm the absence of GI organic diseases.

Higher FC values in healthy infants have been explained by an increased immunological stimulation in the pediatric population by antigens against the intestinal mucosa [20], as well as by increased gastrointestinal leakage [13].

Conversely, a series of hypotheses have been proposed trying to explain a potential physiological increase in FC values in the elderly, yet without definitive conclusions. Aging has been associated with gut dysbiosis and an impaired intestinal barrier and motility. Coupled with gut immunosenescence, lifestyle changes, and a generally worsened health status, these factors may lead to chronic low-grade inflammation, known as “inflammaging”, also at the intestinal level [15,16,21]. For instance, Dunlop et al. demonstrated a significant decrease in lamina propria T-lymphocyte counts, crypt intraepithelial T lymphocyte, and mast cells in the population over 55 years old, possibly due to senescence mechanisms such as reduced T cell progenitors, thymic involution, altered activation pathways, or the reduced gut-homing of circulating T cells [21]. Therefore, advancing age may be associated with compromised gut mucosal integrity, resulting in an amplified immune response to antigenic stimuli [15], despite the reduced numbers of mucosal inflammatory cells.

In our work, we also observed globally higher median FC values in the older group compared to the younger subjects. Nevertheless, our findings indicated that FC showed reliable and comparable diagnostic accuracy in distinguishing organic-inflammatory GI diseases from functional GI disorders, with relevant sensitivity and specificity, both in younger and older groups.

In addition, higher FC levels have been reported in different extraintestinal conditions, such as rheumatic, dermatological, and neurological diseases [22,23,24]. In particular, growing evidence recognizes the role of the “gut–brain axis” in the pathogenesis of some neurologic disorders, mainly Parkinson’s and Alzheimer’s diseases, which represent typical conditions of advanced age. Increased levels of FC have been found in patients with Parkinson’s disease in respect to controls, interestingly with a significant between-group difference only in older subjects (i.e., ≥61 years) [24].

As expected, our older subjects showed higher values of the Charlson Comorbidity Index with respect to younger subjects. This result may contribute to explaining the significant difference in global median values of FC between the two groups and, specifically, the slight increase in median FC values over the range of 50 μg/g (72 μg/g) in the elderly.

Interestingly, the best cut-offs to discriminate between organic-inflammatory GI diseases and functional GI disorders were quite similar in both age groups.

Although the threshold of 50 μg/g represented the most commonly adopted cut-off FC value to discriminate between “normal” and “abnormal” results, as also suggested by most kit manufacturers, there is still a lack of agreement on the best cut-off levels in FC. Different threshold concentrations have been proposed for differentiating IBD from IBS, for predicting endoscopic activity, remission, and relapse, for discriminating organic-inflammatory GI diseases vs. functional GI disorders, mainly due to the heterogeneity of the study population [14,25,26,27]. Previously, *Von Roon* et al. reported in their meta-analysis that the cut-off of 100 μg/g was better than 50 μg/g for differentiating patients with IBD from those with IBS, with a specificity of 0.91 (95% CI 0.86–0.91), and sensitivity of 0.95 (95% CI 0.93–0.97) (24). More recently, the cut-off of ≤50 μg/g showed the best combination of sensitivity and specificity in differentiating IBD from IBS patients [14]. In addition, the meta-analysis by Carrasco-Labra et al. evaluated the performance of this marker in distinguishing patients with organic diseases (also including IBD) from those with functional diarrhea or IBS, showing a sensitivity and specificity of fecal calprotectin, at a cut-off of 50–60 μg/g, of 81% and 87%, respectively [11].

All these systematic reviews included studies involving various age ranges of patients; however, a focused analysis among different age subgroups was still lacking.

As for Carrasco-Labra’s meta-analysis, it is conceivable that, although most patients with organic diseases had IBDs, the inclusion of other organic conditions in the final analysis may be responsible for a slight decrease in the sensitivity and specificity of fecal calprotectin at the standard cut-off of 50 μg/g. By our ROC analysis, a cut-off of 100 μg/g could be suggested as the best threshold level to discriminate organic-inflammatory GI diseases vs. functional GI disorders independently from the age subgroups. Excluding IBDs from the analysis, we found quite low FC thresholds to be suggested, closest to 50 μg/g, especially in the older group, with a remarkable specificity. Conversely, in the younger group, the threshold FC levels remained close to 100 μg/g. As reported above, we only included subjects with at least one year of follow-up or with a documented final diagnosis. However, it is possible that a variable degree of subclinical intestinal inflammation, expressed by the moderate increase in FC values, can be found in a subgroup of younger subjects not achieving a prompt diagnosis of inflammatory bowel disease, rather deserving a careful, and maybe longer, follow-up.

It may be surprising that, in our study, the prevalence of organic-inflammatory GI diseases was higher in young patients, as evidenced by Table 3. Although it is known that IBD incidence is overall increasing in the elderly, in our cohort, most of the patients undergoing an FC assay, and receiving a final diagnosis of IBD, were aged <65 years. In any case, when examining all other organic-inflammatory diseases excluding IBD, it becomes evident that their occurrence is more prevalent among the older group. In fact, there are other known intestinal inflammatory conditions also showing a moderate increase in FC over the normal threshold, such as diverticulitis, infectious colitis, nonsteroidal anti-inflammatory drugs (NSAID) enteropathy, colorectal cancer, that are usually associated with increasing age [3,4,9,10,11].

In addition to the indisputable role in the management of known cases of IBDs, one of the main benefits of FC in clinical practice could be represented by the identification of adult patients requiring further invasive investigations, such as endoscopic examinations, in the search for intestinal inflammation. The meta-analysis by *van Rheenen* et al. demonstrated that screening with FC could reduce unnecessary colonoscopies by 67% in those suspected of having IBD [26]. Similar results were documented by *Mindemark and colleagues*, with a reduction in colonoscopies by 50% using the FC cut-off of <50 μg/g and 67% using the FC cut-off of <100 μg/g [27]. These results are of great importance in older patients, since colonoscopy represents an invasive examination and is burdened by a greater risk of complications in comparison to younger subjects.

In any case, it has to be highlighted that FC represents a poor test for colorectal cancer and is not indicated as a screening test for this condition. Instead, in the absence of alarming symptoms, we suggested that its assay might be encouraged, particularly in older populations, to rule out organic-inflammatory GI diseases, since the specificity remains very high, especially at similar threshold levels compared to younger subjects.

Our study has some limitations. Firstly, the retrospective design of this work could have limited the homogeneity of sample study. However, a review of all available records was performed by the same member of the staff, excluding all those subjects without a definitive and documented final diagnosis within a year. Secondly, the time of follow-up was limited to one year. It is possible that, specifically for the younger group, this time was not enough for a small percentage of the enrolled subjects to identify any possible case of IBD or other inflammatory GI diseases. Moreover, our cohort consisted of subjects (mostly young patients with a known diagnosis of IBD) undergoing FC assays in a tertiary center, most of whom following the advice of gastroenterologists and other specialists; therefore, these results cannot be generalized to a primary care clinical scenario where the prevalence, especially of IBDs, may be substantially different. More than half of patients with an FGID diagnosis was included in the group of unspecified functional bowel disorders, as a result of medical record review. However, we cannot rule out the possibility, for some of these subjects, to meet criteria for another specified FGID during follow-up. It is likely that larger prospective studies could help in providing a more precise classification. Finally, we did not record medications, including NSAIDs, taken by our subjects at the time of the FC assay, since these data were lacking for the most of the enrolled patients in the available records. In light of these limitations, it will be interesting to further validate the results through subsequent prospective studies, and it is likely that larger investigations may provide more evidence about the usefulness and reliability of FC in a geriatric overall context as well.

## Figures and Tables

**Table 1:** Main demographic, clinical, and laboratory features of the total cohort and different subgroups according to age.

		Age		
	Total Cohort n. 745	<65 Years n. 573	>65 Years n. 172	*p*
Demographic and clinical features				
Median age (years)	48 (33–63)	42 (29–53)	74 (68–78)	<0.01
Sex (females, %)	429 (57.6%)	335 (58.5%)	94 (54.7%)	0.38
Charlson Comorbidity Index	1 (0–3)	0 (0–0)	4 (3–6)	<0.01
White blood cell count (10^9/L)	6.9 (5.2–9.1)	6.8 (5.2–9.1)	7.1 (5.2–9.5)	0.60
C-reactive protein (mg/L)	8.5 (1.7–35.0)	6.4 (1.4–24.5)	18.8 (4.4–73.9)	<0.01
Fecal calprotectin (µg/g)	49 (25–185)	47 (25–165.5)	72 (25–260)	<0.01
Indications to undergo FC assay				
Diarrhea	194 (26.%)	144 (25.1%)	50 (29.1%)	0.30
Abdominal pain	521 (69.9%)	405 (70.7%)	116 (67.4%)	0.42
Rectal bleeding	10 (1.3%)	8 (1.4%)	2 (1.2%)	0.82
IBD follow-up	92 (12.3%)	75 (13.1%)	17 (9.9%)	0.26
Main final diagnosis				
IBD	244 (32.8%)	204 (35.6%)	40 (23.3%)	<0.01
Active IBD	167 (22.4%)	139 (24.3%)	28 (16.3%)	0.28
Colorectal cancer and high-risk polyp	9 (1.3%)	3 (0.5%)	6 (3.5%)	<0.01
Acute bowel inflammation (non-IBD)	6 (0.8%)	10 (0.2%)	6 (3.5%)	0.22
Acute diverticulitis	14 (1.9%)	2 (0.3%)	12 (7.0%)	<0.01
Infective colitis	40 (5.4%)	22 (3.8%)	18 (10.5%)	<0.01
Ischemic colitis	2 (0.3%)	1 (0.2%)	1 (0.6%)	0.41
Symptomatic UDD	10 (1.3%)	4 (0.7%)	6 (3.5%)	0.01
Upper endoscopic pathological findings	12 (1.6%)	7 (1.2%)	5 (3.0%)	0.06
Uncomplicated hemorrhoids	44 (5.9%)	28 (4.9%)	18 (10.5%)	0.01
Asymptomatic diverticulosis	78 (10.5%)	34 (5.9%)	44 (25.6%)	0.65
Non-adenomatous polyp	46 (6.2%)	20 (3.5%)	26 (15.1%)	<0.01
Specified FGIDs	194 (22.0%)	142 (24.8%)	52 (30.2%)	0.05
Unspecified FBDs	295 (39.6%)	234 (40.8%)	61 (35.5%)	0.06

IBD: inflammatory bowel diseases. FC: fecal calprotectin. UDD: uncomplicated diverticular disease. FGIDs: functional gastrointestinal disorders (IBS, functional constipation, functional diarrhea, or functional bloating with or without distension); FBDs: functional bowel disorders. Indications to undergo an FC assay and main final diagnosis are expressed by number of patients and percentage.

**Table 2:** Main demographic, clinical and laboratory features of total cohort according to final organic-inflammatory GI diseases vs. functional GI disorders.

	Organic-Inflammatory GID n. 256	Functional GID n. 489	*p*
Demographic and clinical features			
Median age (years)	50 (32–66.75)	48 (35–62)	0.31
Age > 65 years (n. of patients, %)	74 (28.9%)	98 (20%)	0.01
Sex (females, %)	144 (56.3%)	285 (58.3%)	0.59
Charlson Comorbidity Index	1 (0–3)	1 (0–2)	0.03
White blood cell count (10^9/L)	7.7 (5.9–10.8)	6.5 (5.0–8.6)	<0.01
C-reactive protein (mg/L)	11.5 (2.9–47.7)	6.6 (1.4–28.1)	<0.01
Fecal calprotectin (µg/g)	365 (163.3–1090)	34 (18–50)	<0.01
Indications to undergo FC assay			
Diarrhea (n. of patients, %)	126 (49.2%)	68 (13.9%)	<0.01
Abdominal pain (n. of patients, %)	144 (56.3%)	377 (77.1%)	<0.01
Rectal bleeding (n. of patients, %)	10 (3.9%)	0 (0.0%)	<0.01

GID: gastrointestinal diseases. FC: fecal calprotectin. Indications to undergo an FC assay and main final diagnosis are expressed by number of patients and percentage.

**Table 3:** Sensitivities, specificities, and positive and negative predictive values for FC in discriminating organic-inflammatory GI diseases vs. functional GI disorders in the total cohort and in the two different age subgroups at different thresholds.

FC Threshold (μg/g)	Sensitivity (95% CI)	Specificity (95% CI)	PPV (95% CI)	NPV (95% CI)
Total cohort				
≤50	77.5 (73.5–81.1)	95.3 (92.0–97.6)	96.9 (94.8–98.2)	68.9 (65.2–72.4)
≤100	94.5 (92.1–96.3)	85.6 (80.6–89.6)	92.6 (90.3–94.4)	89.0 (84.9–92.2)
≤102	95.1 (92.8–96.8)	85.2 (80.2–89.3)	92.4 (90.1–94.2)	90.1 (86.0–93.1)
Age < 65 years				
≤50	78.8 (74.4–82.7)	93.4 (88.8–96.5)	96.2 (93.7–97.8)	67.2 (62.8–71.3)
≤100	94.9 (92.2–96.8)	86.8 (81.0–91.4)	93.9 (91.4–95.7)	89.3 (84.2–92.8)
≤109	95.9 (93.4–97.6)	86.3 (80.4–90.9)	93.7 (91.2–95.6)	90.8 (85.8–94.1)
Age ≥ 65 years				
≤50	72.5 (62.5–81.1)	98.7 (92.7–100.0)	98.6 (91.2–99.8)	73.0 (66.2–78.9)
≤98	93.9 (87.1–97.7)	82.4 (71.8–90.3)	87.8 (81.2–92.1)	91.0 (82.3–95.7)
≤100	94.9 (88.5–98.3)	81.1 (70.3–89.3)	86.9 (80.5–91.4)	92.3 (83.5–96.6)

FC: fecal calprotectin. GI: gastrointestinal. PPV: positive predictive value. NPV: negative predictive value.

**Table 4:** Main demographic, clinical, and laboratory features of the total cohort, not including IBDs, according to diagnosis of organic-inflammatory GI diseases vs. functional GI disorders.

	Organic-Inflammatory GID n. 85	Functional GID n. 416	*p*
Demographic and clinical features			
Median age (years)	65 (43–78)	48 (32–62)	<0.01
Age > 65 years (n. of patients, %)	43 (50.6%)	89 (21.4%)	<0.01
Sex (females, %)	35 (41.2%)	176 (42.3%)	0.85
Charlson Comorbidity Index	3 (0–5)	1 (0–3)	<0.01
White blood cell count (10^9^/L)	7.1 (5.2–9.9)	6.5 (5.0–8.3)	0.07
C-reactive protein (mg/L)	15.3 (2.9–72.7)	7.6 (1.5–33.9)	*0.03*
Fecal calprotectin (µg/g)	248 (81–500)	35 (18–50)	<0.01
Diarrhea (n. of patients, %)	31 (36.5%)	68 (16.3%)	<0.01
Abdominal pain (n. of patients, %)	57 (67.1%)	376 (90.4%)	<0.01
Rectal bleeding (n. of patients, %)	3 (3.5%)	0 (0.0%)	<0.01

GID: gastrointestinal diseases. FC: fecal calprotectin. Indications to undergo an FC assay and main final diagnosis are expressed by number of patients and percentage.

**Table 5:** Sensitivities, specificities, and positive and negative predictive values for FC in discriminating organic-inflammatory GI diseases (not including IBDs) vs. functional GI disorders in the total cohort and in the two different age subgroups at different thresholds.

FC Threshold (μg/g)	Sensitivity (95% CI)	Specificity (95% CI)	PPV (95% CI)	NPV (95% CI)
Total cohort				
≤50	76.2 (71.8–80.2)	89.4 (80.8–95.0)	97.2 (95.0–98.5)	43.4 (38.9–48.1)
≤70	84.4 (80.5–87.7)	82.4 (72.6–89.8)	95.9 (93.7–97.4)	51.9 (45.8–57.9)
≤100	93.8 (91.0–95.9)	70.6 (59.7–80.0)	94.0 (91.8–95.8)	69.8 (60.8–77.4)
Age < 65 years				
≤50	78.5 (74.1–82.4)	94.4 (90.4–97.1)	96.3 (93.8–97.9)	70.5 (66.4–74.4)
≤100	95.0 (92.4–96.9)	87.3 (82.1–91.5)	93.4 (90.8–95.2)	90.3 (85.8–93.5)
≤102	95.3 (92.7–97.1)	87.3 (82.1–91.5)	93.4 (90.8–95.3)	90.7 (86.3–93.8)
Age ≥ 65 years				
≤45	73.0 (62.6–81.9)	100.0 (91.8–100.0)	100 (92.0–100.0)	64.2 (56.0–71.6)
≤50	73.0 (62.6–81.9)	97.7 (87.7–99.9)	98.5 (90.3–99.8)	63.6 (55.3–71.2)
≤100	94.4 (87.4–98.2)	74.4 (58.8–86.5)	88.4 (82.1–92.7)	86.5 (72.8–93.9)

GI: gastrointestinal FC: fecal calprotectin. PPV: positive predictive value. NPV:negative predictive value.

## Data Availability

The data presented in this study are available on request from the corresponding author.
